# Actual and perceived motor competence: Are children accurate in their perceptions?

**DOI:** 10.1371/journal.pone.0233190

**Published:** 2020-05-13

**Authors:** Milena Morano, Laura Bortoli, Montse C. Ruiz, Angelo Campanozzi, Claudio Robazza

**Affiliations:** 1 Parisi-De Sanctis Institute, MIUR (Italian Ministry of Education, University and Research), Foggia, Italy; 2 School of Medicine and Health Sciences, “G. d’Annunzio” University of Chieti-Pescara, Chieti, Italy; 3 BIND-Behavioral Imaging and Neural Dynamics Center, Department of Medicine and Aging Sciences, “G. d’Annunzio” University of Chieti-Pescara, Chieti, Italy; 4 Faculty of Sport and Health Sciences, University of Jyväskylä, Jyväskylä, Finland; 5 Pediatrics, Department of Medical and Surgical Sciences, University of Foggia, Foggia, Italy; University of Rome, ITALY

## Abstract

The aims of this study were (1) to investigate whether 6−7-year-old children are accurate in perceiving their actual movement competence, and (2) to examine possible age- and gender-related differences. A total of 603 children (301 girls and 302 boys, aged 6 to 7 years) were assessed on the execution accuracy of six locomotor skills and six object control skills using the Test of Gross Motor Development (TGMD-2). The perceived competence of the same skills, plus six active play activities, was also gauged through the Pictorial Scale of Perceived Movement Skill Competence (PMSC-2). The factorial validity of the TGMD-2 and PMSC-2 scales was preliminarily ascertained using a Bayesian structural equation modeling approach. The relationships between the latent factors of the two instruments were then assessed. Gender and age differences were also examined. The factorial validity of the TGMD-2 and the PMSC-2 was confirmed after some adjustments. A subsequent analysis of the relationship between the latent factors (i.e., locomotor skills and object control) of the two instruments yielded very low estimates. Finally, boys and older children showed better competence in object control skills compared to their counterparts. Weak associations between actual and perceived competence suggest that inaccuracy in children’s perceptions can be likely due to a still limited development of cognitive skills needed for the evaluation of the own competence. From an applied perspective, interventions aimed at improving actual motor competence may also increase children’s self-perceived motor competence and their motivation toward physical activity.

## Introduction

The development of motor competence in children and adolescents and the adoption of a lifestyle involving participation in regular exercise and physical activity have been associated with numerous physical and psychological health benefits, which are well documented in the literature [1–3]. Motor competence can be defined as a person’s ability to execute a wide range of motor acts in a proficient manner, including coordination of fine and gross motor skills that are necessary to manage everyday tasks, such as walking, running, jumping, catching, throwing, kicking, and rolling [4–6]. It is considered one of the most powerful underlying mechanisms that promote engagement and persistence in physical activity, higher levels of sport participation, and physical fitness [2,3]. Therefore, children with a higher level of actual motor competence are more apt to become physically active and fit adolescents [3]. Research has shown that the relationship between physical activity and motor competence might vary depending on children’s age [4].

Together with actual motor competence, perceived competence—that is, an individual’s perception of their actual abilities [7]—is considered a primary motivational factor underlying voluntary participation in any sport or physical activity [8]. Harter [9] proposed actual competence to be an antecedent of perceived competence, which influences physical activity and is a strong determinant of a child’s motivation. Experiences of successful motor executions, measured in terms of increased skill proficiency, are expected to enhance perceived competence, which in turn might influence motivation toward physical activity [10]. Hence, both actual and perceived motor competence are considered important correlates and determinants of physical activity and fitness in boys and girls [5].

Fundamental motor skills are commonly considered the building blocks for more advanced lifetime movement skills and the foundation for an active lifestyle [10,11]. Previous research has demonstrated the existence of age and gender differences in mastery of fundamental motor skills [12,13]. These include locomotor skills, involving the movement of the body from one location to another (e.g., running and hopping); object-control skills, including the manipulation of an object (e.g., catching and throwing); and stability skills/body management (e.g., balancing and twisting; [11]). Higher fundamental motor skill competence was found with increasing age [12,13] as a result of learning and practice [14]. Furthermore, object control skills appeared to be better mastered by boys [11–13,15], who also reported higher perceived competence scores compared to girls [16,17]. Gender differences were also reported by Pesce et al. [18] who assessed both actual and perceived fundamental motor skill competence in Italian children aged 7.5±1.2 years. Findings showed that about 30% of children underestimated or overestimated their locomotor and object control skills. The percentage of girls who underestimated their skills was higher than the percentage of girls who overestimated themselves. In boys an opposite trend was observed with a higher percentage of overestimators. Interestingly, children who overestimated their locomotor competence were also found to practice a larger amount of sport than those who underestimated themselves.

However, differences between actual and perceived motor competence in young children are equivocal, probably because existing research in this area has evaluated these two variables with no direct alignment between assessments [17,19], alignment that would derive from measuring actual and perceived movement competence in same skills. To date, few studies have used objective measures of both perceived and actual competence of the same skills [17,18,20,21]. Using aligned assessments (e.g., perceived jumping skill and actual jumping skill assessment) and valid and reliable measures can help researchers to appropriately monitor actual and perceived motor competence in childhood, starting in the early years. Different measures have been developed and validated to evaluate actual and perceived fundamental motor skills in children. For instance, the Test of Gross Motor Development– 2nd edition (TGMD-2; [22]), intended to measure motor competence in 3 to 10 years old children, is one of the most widely used instruments in clinical, educational, and research settings. In order to understand how children’s perceptions relate to actual movement competence, Barnett and colleagues [23–25] developed the pictorial scale of Perceived Movement Skill Competence– 2nd edition (PMSC-2) assessing the same 12 skills included in the TGMD-2 [22] and 6 additional skills (i.e., active play activities).

Until recently, however, no assessment instrument was available to reliably gauge children’s actual execution of motor skills and perceived motor competence of the same skills [17]. Given the importance of assessing actual and perceived motor skill competence in young children, the aim of our study was to investigate whether actual motor competence of 6-7-year-old children related to their perceptions of competence. The link between actual and perceived competence in the same skills using the TGMD-2 and PMSC-2 scales was previously examined by Pesce et al. [18] in an Italian sample of children. As previously noted, results showed that a substantial percentage of children underestimated or overestimated their locomotor and object control skills. However, the factorial validity of the two scales was not examined before conducting the analyses. Therefore, further investigation is needed to examine the extent to which children are able to accurately evaluate themselves. To this purpose, we first examined the factorial validity of the TGMD-2 and PMSC-2 scales, and then the relationships among the latent factors of the two scales. In line with earlier findings [18], we expected to find weak associations between actual and perceived competence in children. A secondary purpose of this study was to investigate possible age- and gender-related differences. We expected boys and older children to show higher levels of both actual and perceived skill competence than girls and the younger cohort, respectively [4,13,18].

## Method

### Participants

The initial sample comprised 603 children (301 girls and 302 boys), aged 6 to 7 years, drawn from 36 mixed gender classes of primary schools located in a region in Central Italy. At the time of the assessment the participants did not suffer from visible diseases, were able to take part in school physical activities, and did not have diagnosed physical or cognitive impairments (the latter information was certified by the teachers). The assessment was part of a larger project named “Increase in physical activity in I and II classes of Primary School”, which aimed to prevent obesity and promote healthy lifestyles in children through physical activity conducted by expert physical education teachers during customary lessons (see [26]).

### Measures

#### Test of Gross Motor Development (TGMD-2)

The TGMD-2 is a process-oriented assessment tool designed to gauge the gross motor development of 3 to 10 years old children [22]. The test consists of two six-item subscales to measure locomotor skills (i.e., running, galloping, hopping, leaping, jumping, and sliding) and object control skills (i.e., striking, dribbling, catching, kicking, throwing, and underhand rolling a ball). Specific guidelines for completion of the TGMD-2 exist [22]. Each skill is explained and demonstrated to the children by trained assessors using standard instructions. The participant is then allowed one practice trial followed by two formal trials that are scored according to 3 to 5, behaviorally-based performance criteria, depending on the skill. Participants are assigned a score of ‘1’ if they perform correctly a performance criterion, or a score of ‘0’ if they execute incorrectly, resulting in a maximum score of 3, 4, or 5 for each skill depending on the number of performance criteria in each skill. A total raw score (0–48 points) is the sum of the observed criteria for each item of the two subscales. The individual assessment can usually be completed within 20–30 min. All trials are videotaped, and coding is conducted through video analysis. Research findings generally support the two-factor structure, validity, and reliability of the TGMD-2 (e.g., [6,27–29]; for methodological issues related to validation research of the TGMD-2 see [30]).

#### Pictorial Scale of Perceived Movement Skill Competence (PMSC-2)

Barnett and colleagues [23–25] developed the PMSC-2 pictorial scale assessing the same six locomotor and six object control skills included in the TGMD-2 [22]. Six additional items assess perceived competence in active play related to free time activities (riding a bike, riding a scooter, lying on a board and paddling with the arms, roller skating, swimming, climbing a rope). Each skill is ordered in a sequence of cartoon images of a child executing the skill competently opposed to an image of a child executing the same skill not so competently. Thus, two pictures depict good and poor performance of a same skill. Girls are presented with a booklet portraying girl cartoon figures, while boys are presented with boy figures. Children are required to choose which picture is most like them (i.e., ‘this child is pretty good at swimming, this child is not that good at swimming, which child is like you?’). Then, within the chosen picture, children are asked to further indicate their perceived competence. If children select the competent picture they are asked: ‘are you really good at …’ (score of four) or ‘pretty good at …’ (score of three). If children choose the not so competent picture, they are asked: are you ‘not that good at (score of one) or sort of good at…’ (score of two). Thus, four perceived competence levels are assessed in each picture, with possible scores ranging from 6 to 24 in each subscale. A total raw score (0–72 points) is the sum of perceived competence scores for each item of the three subscales. The individual assessment usually takes approximately 15–20 min. Reliability and validity of the instrument have been established [23–25]. For example, in a sample of Australian children alpha reliability values were .84, .76, and .78 for locomotor, object control, and active play skills, respectively [25]. Factor loadings ranged from .41 to .83.

### Procedure

Ethical approval for the study was granted from the Health Department of the Abruzzo Region in reference to the Regional Prevention Plan 2014–2018—Program 2, Action 2. Approval was also obtained from the school headmasters after the purpose of the study was explained to them. All participants’ parents or guardians signed a written informed consent form with anonymity and confidentiality being assured. Four experts were specifically instructed in administering the TGMD-2 and coding the data. The TGMD-2 assessment was conducted in pairs, with two evaluators always present. One evaluator conducted the actual assessment, including providing instructions and visual demonstration, while the other digitally recorded the child’s performance. Each child was allowed one practice trial before two formal trials. Each evaluator then rated independently the video recorded performances. In case of disagreement, the video recorded performance was reexamined to reach consensus (see Results). Other three experts were instructed about administration and scoring of the PMSC-2 scale. TGMD-2 assessments were conducted individually during physical education lessons at a shared gym space but separate from the usual activity sessions held by the physical education teacher. PMSC-2 assessments were also conducted individually in a secluded location close to the gym and without the presence of the teacher. Children were presented with the booklet depicting the skills to be assessed, informed that there were no right or wrong responses, and assured that their answers would remain confidential. The evaluators always made sure that children had complete understanding of the instructions and items of both instruments.

### Bayesian data analysis

Muthén and Asparouhov [31] advocated Bayesian analysis as an alternative approach to factor analysis and structural equation modeling (SEM) frequentist methods. They argued that analyses using maximum-likelihood (ML) and likelihood-ratio chi-square (χ^2^) testing apply unreasonably stringent models to represent hypotheses derived from substantive theory. Fixing parameters to zero (e.g., zero cross-loadings and zero residual correlations) in factor analysis often leads to rejection of the model and subsequent model modifications that may capitalize on chance, as the respecifications may reflect idiosyncratic characteristics of the sample. As a result, the improved fit of the modified model in a particular sample may not replicate in another sample. Bayesian models are more flexible allowing parameter estimation even with small sample sizes where SEM approaches often lead to model identification issues. Moreover, Bayesian approaches allow the incorporation of previous knowledge into the analyses, which can provide more precise estimates of the parameters in the model tests [32,33]. In particular, Bayesian structural equation modeling (BSEM), as an alternative to SEM, can better reflect substantive theories by replacing the parameter specification (e.g., cross-loadings and residual correlations) of exact zeros with parameter values estimated via prior distributions (hyperparameters). Prior distributions can be informative priors based on previous findings and theoretical predictions, or empirical priors based on observed data [33].

We conducted BSEM with the M*plus* 8.4 statistical software [34] using Markov Chain Monte Carlo (MCMC) simulation process using the Gibbs’ algorithm [35] initially with 50,000 iterations and then 100,000 to check convergence and the stability of the estimates. MCMC involves an iterative process where a prior distribution is specified, and posterior values of each parameter are estimated in a chain of a number of iterations to define the posterior distribution. Convergence of the iteration process can be evaluated through the potential scale reduction (PSR), which represents the ratio of total variance between chains and pooled variance within chain. An acceptable convergence level is reached when PSR is < 1.10 (a PSR of 1.00 is considered perfect model convergence; [36]). Convergence can also be assessed by visually inspecting trace and autocorrelation plots of the posterior distribution. Convergence is manifested when fluctuations in the chain and trends over time of generated parameter values are stable [37].

First, we performed BSEM to examine the factorial validity of the TGMD-2 and PMSC-2 scales. To improve the structural solution, the first step was to conduct a pilot study in a small sample randomly extracted from the whole sample from which the prior information was obtained [38]. Second, to examine possible gender and age differences in the latent factors on both scales, gender and age (i.e., 6 and 7 years) were dummy coded to represent group membership and entered as covariates in the models. A gender by age interaction term was also computed and entered as covariate. Third, we conducted BSEM on the full model to assess the relationships between the latent factors of the TGMD-2 (i.e., locomotor and object control) and the latent factors of the PMSC-2 (i.e., locomotor, object control, and active play). Items of both scales were treated as categorical rather than continuous variables because the item ratings of the TGMD-2 ranged from zero to three, four, or five points, and the PMCS-2 items were four-point ordinal responses.

Fit of the BSEM models was assessed using two recommended criteria based on the goodness-of-fit χ^2^: (a) the 95% confidence interval comparing the difference between the observed data and the replicated χ^2^ values, and (b) the resulting posterior predictive *p*-value (PPP). A low PPP (*p* < .05) and a positive 95% lower limit indicate poor model fit, whereas PPP values exceeding .05 and around .50 and a symmetric 95% confidence interval centered around zero imply good model fit [31].

## Results

### Preliminary analysis

The initial data screening led to the removal of 14 cases identified as multivariate outliers using Mahalanobis’ distance (*p* < .001). The final sample comprised 589 children (123 girls and 119 boys, 6 years old; 172 girls and 175 boys, 7 years old; see S1 Dataset). Interrater agreement between the TGMD-2 evaluators was high, with intraclass correlation (ICC) values of .825 (95% C.I. = .796–.850) for locomotor skills, and .925 (95% C.I. = .913–.936) for object control skills.

A subsample of 180 children, with an equal number of participants (i.e., 45) by gender and age, was randomly selected from the whole sample to identify empirical priors to be used in the subsequent analysis of the large sample of 409 children (78 girls and 74 boys, 6 years old; 127 girls and 130 boys, 7 years old). Convergence in the subsample was reached on both the TGMD-2 and PMSC-2 data (PSR < 1.01) and the visual inspection of plots for each of the model parameters was consistent with that finding. Model fit was satisfactory for the TGMD-2, PPP = .590, 95% CI χ^2^ = -41.214, 31.505, and acceptable for the PMSC-2, PPP = .066, 95% CI χ^2^ = -12.222, 101.689.

### Factorial validity of the TGMD-2 and PMSC-2 scales

On the large sample (*n* = 409), BSEM without informative priors on the TGMD-2 data yielded acceptable model fit PPP = .256, 95% CI χ^2^ = -24.230, 48.371. On the other hand, the PMSC-2 data did not fit the expected model, PPP = .010, 95% CI χ^2^ = 11.490, 124.810. The PMSC-2 data were reassessed using informative priors, namely, cross-loading parameter values previously estimated on the pilot study. Three problematic items, one for each factor, showed poor factor loadings (≤ .250) in the appropriate factor and significant cross-loadings in other factors. Three items, namely, sliding (locomotion factor), rolling a ball (object control factor), and riding a scooter (active play factor) were then removed leading to a three-factor, 15-item scale (5 items in each factor).

Subsequent analyses on the large sample involved testing the goodness-of-fit on a model without priors, a model with informative priors on cross-loadings, and a model with informative priors on cross-loadings and residual correlations within an identified model. BSEM fit and convergence results reported in Table 1 show model fit improvement when informative priors (cross-loadings and residual correlations) were included in the analysis. Standardized factor loadings of items with 95% confidence intervals are reported in Table 2.

**Table 1:** BSEM fit and convergence (*n* = 409).

Model	N. of free parameters	PPP	Lower CI χ^2^	Upper CI χ^2^	PSR
TGMD-2					
Non-informative	57	.256	-24.230	48.371	1.00
Informative priors (cross-loadings)	69	.332	-29.496	42.853	1.00
Informative priors (cross-loadings and residual correlations)	135	.585	-41.311	33.134	1.01
PMSC-2 (15 items)					
Non-informative	75	.010	11.490	124.810	1.00
Informative priors (cross-loadings)	111	.094	-18.687	92.992	1.00
Informative priors (cross-loadings and residual correlations)	264	.629	-62.439	44.284	1.01

PPP = posterior predictive *p*-value; Lower CI χ^2^ = lower confidence interval for the difference between the observed and the replicated chi-square values; Upper CI χ^2^ = upper confidence interval for the difference between the observed and the replicated chi-square values; PSR = potential scale reduction.

**Table 2:** BSEM model solutions using informative priors for cross-loadings and residual correlations (*n* = 409).

Item	TGMD-2		PMSC-2 (15 items)		
	Locomotor	Object control	Locomotor	Object control	Active play
Running	**.457 [.301, .619]**	.004 [-.135,.144]	**.474 [.312, .623]**	.025 [-.138, .174]	-.006 [-.159, .139]
Galloping	**.347 [.071, .663]**	.004 [-.152, .154]	**.499 [.196, .797]**	-.005 [-.175, .203]	-.007 [-.219, .195]
Hopping	**.732 [.507, .917]**	.000 [-.100, .099]	**.627 [.333, .829]**	.005 [-.149, .185]	.005 [-.148, .157]
Leaping	**.380 [.087, .641]**	.003 [-.143, .147]	**.651 [.304, .871]**	.025 [-.129, .198]	.028 [-.103, .172]
Jumping	**.585 [.30, .831]**	.003 [-.106, .118]	**.584 [.320, .845]**	.006 [-.159, .182]	-.002 [-.150, .185]
*Sliding	**.593 [.317, .841]**	.004 [-.108, .116]			
Striking	-.003 [-.159, .156]	**.345 [.182, .557]**	.051 [-.141, .262]	**.393 [.260, .624]**	-.005 [-.177, .147]
Dribbling	.023 [-.141, .193]	**.401 [.045, .818]**	.016 [-.194, .224]	**.394 [.017, .696]**	.004 [-.167, .186]
Catching	.039 [-.12, .210]	**.393 [.021, .701]**	.024 [-.172, .234]	**.550 [.212, .815]**	-.010 [-.192, .167]
Kicking	-.002 [-.167, .165]	**.390 [-.025, .708]**	.005 [-.220, .224]	**.540 [.159, .822]**	.002 [-.174, .168]
Throwing	.028 [-.141, .213]	**.430 [-.016, .760]**	.014 [-.167, .184]	**.677 [.231, .933]**	.047 [-.084, .343]
*Rolling a ball	-.005 [-.167, .177]	**.507 [.022, .834]**			
Riding a bike			.040 [-.186, .259]	-.023 [-.197, .146]	**.466 [.277, .640]**
*Riding a scooter					
Paddling on a board			.032 [-.136, .194]	.032 [-.110, .216]	**.657 [.240, .871]**
Roller skating			-.062 [-.259, .138]	-.027 [-.213, .134]	**.581 [.325, .837]**
Swimming			.046 [-.162, .238]	.017 [-.145, .211]	**.472 [.052, .754]**
Climbing a rope			.024 [-.147, .197]	.019 [-.111, .199]	**.689 [.429, .946]**

Standardized factor loadings with 95% confidence intervals in brackets. Loadings and 95% confidence intervals on intended factors are highlighted in bold text.

*PMSC-2 items removed from further analyses.

### Relationships between latent factors

BSEM with informative priors on cross-loadings and residual correlations on the whole model (i.e., both TGMD-2 and PMSC-2 15-item data) did not reach convergence. Analysis with informative priors on cross-loadings reached convergence and yielded acceptable fit PPP = .134, 95% CI χ^2^ = -33.398, 122.059. Standardized estimates between the latent factors of the TGMD-2 and the latent factors of the PMSC-2 (15-items) were very low in magnitude (Table 3).

**Table 3:** Standardized estimates and 95% confidence intervals between latent factors (*n* = 409).

Test	TGMD-2		PMSC-2 (15 items)	
Factors	Locomotor	Object control	Locomotor	Object control
TGMD-2				
Locomotor	--			
Object control	.464 [.256, .660]	--		
PMSC-2 (15 items)				
Locomotor	-.001 [-.160, .170]	.077 [-.109, .264]	--	
Object control	-.146 [-.301, .013]	-.090 [-.270, .096]	.513 [.269, .739]	--
Active play	-.155 [-.301, -.001]	-.187 [-.355, -.0171]	.440 [.194, .652]	.650 [.490, .792]

To further explore the relationship between the latent factors of the two instruments, children’s scores of the complete sample (*N* = 589) were partitioned into quartiles of the TGMD-2 total raw scores comprised of the two scales. Quartiles of the PMSC-2 (15-items) total raw scores of the three scales were also derived and participants’ scores placed in a contingency table (Table 4). Similar number of participants distributed in the quartiles of the two instruments was supported by non-significant Pearson χ^2^(9) = 3.436, *p* = .945. This result provides additional evidence of the low relationship between the latent factors of the two measures.

**Table 4:** Number (%) of participants in the whole sample (*N* = 589) classified into quartiles (Q) based on total raw scores of the TGMD-2 and PMSC-2 (15-items).

PMSC-2(15 items)	TGMD-2			
	Q1	Q2	Q3	Q4
Q1	**43 (7.30%)**	51 (8.70%)	34 (5.80%)	37 (6.30%)
Q2	37 (6.30%)	**44 (7.50%)**	25 (4.20%)	36 (6.10%)
Q3	51 (8.70%)	43 (7.30%)	**30 (5.10%)**	35 (5.90%)
Q4	37 (6.30%)	35 (5.90%)	20 (3.40%)	**31 (5.30%)**

Number (%) of participants classified into a same quartile of the two instruments appears in bold text.

### Gender and age differences

BSEM with informative priors cross-loadings and residual correlations, with gender, age, and their interaction as covariate entered in the models, resulted in satisfactory model fit for both TGMD-2, PPP = .561, 95% CI χ^2^ = -47.340, 39.315, and the PMSC-2 (15-items), PPP = .400, 95% CI χ^2^ = -45.638, 59.056. Significant standardized estimates were obtained for object control of the TGMD-2 for gender (.810) and age (.395). Higher scores were observed in boys compared to girls, and in 7-year-old girls and boys compared to 6-year-olds (Table 5).

**Table 5:** Mean (SD) scale scores for the larger sample (*n* = 409).

Age	Gender	TGMD-2		PMSC-2 (15 items)			*n*
		Locomotor	Object control	Locomotor	Object control	Active play	
6 years	Girls	3.23 (0.53)	2.66 (0.53)	3.38 (0.38)	3.32 (0.41)	3.17 (0.54)	78
	Boys	3.25 (0.43)	3.03 (0.48)	3.43 (0.38)	3.32 (0.40)	3.15 (0.60)	74
7 years	Girls	3.38 (0.48)	2.87 (0.45)	3.32 (0.41)	3.12 (0.46)	3.17 (0.52)	127
	Boys	3.38 (0.46)	3.24 (0.38)	3.41 (0.43)	3.31 (0.41)	3.07 (0.57)	130

## Discussion

The main aim of this study was to investigate the relationship between actual and perceived movement competence in 6−7-year-olds. We used the TGMD-2 [22] to measure motor competence, and the PMSC-2 [23–25] to assess children’s perceptions of competence on the same skills contained in the TGMD-2 (i.e., locomotor and object control movements) and six additional activities (i.e., active play). The factorial validity of the TGMD-2 was confirmed in a preliminary analysis, while an acceptable factorial solution of the PMSC-2 was reached after three items were removed, one for each scale. A subsequent analysis of the relationships between the latent factors of the two measures (i.e., locomotor skills and object control) resulted in very low estimates. This finding is similar to the results of some previous studies reporting nonsignificant or weak associations between actual and perceived competence among children in kindergarten and primary school [18,19,39–42]. On the other hand, some of the existing research in this area has demonstrated equivocal findings [16], while other studies have reported moderate associations between perceived physical competence and actual motor skill competence in children [43,44], suggesting that actual and perceived object control competence are more related [17–20,45]. However, it is difficult to compare our findings to those of other studies given that, unlike our study, most prior research investigating children’s actual and perceived motor competence has relied on measures not directly aligned (e.g., [40,44,45]. This is problematic, because the use of unmatched measures of actual and perceived skills can mask their relationship [17].

The lack of association between children’s actual and perceived competence in the present study is in agreement with the notion that young children generally overestimate their actual skills [46] (see mean scores in Table 5), and that until approximately 8 years of age they do not seem to be capable of accurately reporting self-perceptions due to the limited development in their cognition [46–48]. Therefore, given the mean age of the sample in this study, it seems plausible that both girls and boys were not accurate at perceiving their motor competence, because they may not possess the cognitive skills to distinguish between actual motor competence and perceived competence. It is suggested that engagement and efforts to mastery a task build children’s perception of competence. Enhanced perceived competence positively influences children’s effort and continuation in an activity [7], thereby leading to improved actual competence. Thus, perceived competence interacts with actual motor competence strengthening the choice to engage and persist in physical activities [10]. Across developmental time, if children develop sufficient levels of actual motor competence, both the perceptions of competence and the evaluation of personal capabilities can improve and together promote higher levels of physical activity. The use of instruments adapted to the child’s cognition, such as pictorial scales [19,46], can help children align their perceived competence and actual motor competence more closely. From this standpoint, our study contributes to the literature by extending previous research on the relationship between actual and perceived motor competence in 6–7-year-old children. Moreover, the current study is one of few studies using a pictorial scale to assess the perceptions of the same movement skills included in a motor test (e.g., [16–21].

Regarding the gender and age differences found in this study, boys and 7-year-old children showed better competence in object control skills compared to girls and 6-year-olds, respectively. Boys commonly exhibit greater object-control proficiency than girls as a result of different physical activity and activity preferences, with boys participating more in object-control related activities (e.g., ball sports) and girls in locomotor related activities (e.g., dance and gymnastics; see [4] for a systematic review and meta-analysis). The superiority of males in object-control skills can also be attributed to social and environmental factors, as boys enjoy greater encouragement and opportunities for motor experiences through physical activity and sports compared with girls [4]. However, we did not find significant differences in locomotor skills between boys and girls. This is in line with the results of Barnett et al. [4], but in contrast with previous findings among Italian children [49]. According to Clark et al. [50], evidence on gender-related differences in children is still equivocal. Beyond gender, Barnett et al. [4] reported that increasing age is the most consistent correlate of children’s gross motor competence. Older children typically achieve higher levels of fundamental motor skills than their younger counterparts as a result of several contributing factors, including biological maturation and greater exposure to, and practice of, these skills throughout the added years of life [4,13].

## Conclusion

In this study we extended previous research on the relationship between actual and perceived motor competence in 6–7-year-old children. A main strength of the investigation was the alignment between actual and perceived movement skill assessment, which allowed for a better understanding of their relationship, as well as gender and age differences. Another strength was the use of a pictorial instrument to assess perceptions of competence, in order to prompt a straightforward understanding in young children. For applied purposes, our findings highlight that boys and older children outperform girls and their younger counterparts, respectively, in functional movements involving the manipulation of objects, whilst no gender and age differences were found in perceptions of competence. Given that perceived competence is an important motivational factor that influences children’s participation in physical activity and sport [8], teachers and educators are encouraged to implement childhood interventions targeting fundamental movement skills as a strategy for the promotion of long-term physical activity [51]. Specifically, in both school and community settings, special attention should be paid to improving object control skills, given that proficient children in these skills are more likely to become active adolescents [15]. As Duncan et al. [16] suggested, strategies to improve actual motor competence may increase children’s self-perceived motor competence. Thus, providing children with opportunities to succeed and individual encouragement to improve can promote the development of positive and accurate self-perceptions of competence [45]. The relationship between actual and perceived physical competence in children should be further investigated using longitudinal or experimental studies, in order to develop individual skills and attitudes for a long-term physical activity. Additional insight could be gained considering the children’s usual levels and patterns of physical activity outside of school using subjective (e.g., questionnaires and diaries) and objective (e.g., motion sensors and heart-rate monitors) methods.

## Supporting information

S1 Dataset (XLSX)
